# Profiling Activins and Follistatin in Colorectal Cancer According to Clinical Stage, Tumour Sidedness and Smad4 Status

**DOI:** 10.3389/pore.2021.1610032

**Published:** 2021-11-15

**Authors:** Bassem Refaat, Jamal Zekri, Akhmed Aslam, Jawwad Ahmad, Mohammed A. Baghdadi, Abdelrazak Meliti, Shakir Idris, Sufian Sultan, Hosam Alardati, Haitham Akram Saimeh, Aiman Alsaegh, Mai Alhadrami, Tahira Hamid, Mohammed E. Naeem, Shereef Ahmed Elsamany

**Affiliations:** ^1^ Laboratory Medicine Department, Faculty of Applied Medical Sciences, Umm Al-Qura University, Makkah, Saudi Arabia; ^2^ Oncology Department, King Faisal Specialist Hospital and Research Centre, Jeddah, Saudi Arabia; ^3^ College of Medicine, Alfaisal University, Jeddah, Saudi Arabia; ^4^ Research Centre, King Faisal Specialist Hospital and Research Centre, Jeddah, Saudi Arabia; ^5^ Pathology Department, King Faisal Specialist Hospital and Research Centre, Jeddah, Saudi Arabia; ^6^ Department of Laboratory Medicine and Pathology, University of Alberta, Edmonton, AB, Canada; ^7^ Department of Surgery, King Faisal Specialist Hospital and Research Centre, Jeddah, Saudi Arabia; ^8^ Pathology Department, Faculty of Medicine, Umm Al-Qura University, Makkah, Saudi Arabia; ^9^ Histopathology Department, King Abdullah Medical City, Makkah, Saudi Arabia; ^10^ Medical Oncology Department, Oncology Centre, King Abdullah Medical City, Makkah, Saudi Arabia; ^11^ Medical Oncology Department, Oncology Centre, Mansoura University, Mansoura, Egypt

**Keywords:** apoptosis, cell cycle, activin-A, activin-AB, activin-B, follistatin

## Abstract

This study explored the roles of activins and follistatin in colorectal cancers. Paired malignant and normal colonic tissues were collected from archived paraffin-embedded (*n* = 90 patients) alongside fresh (*n* = 40 patients) specimen cohorts. Activin β-subunits, follistatin and Smad4 mRNAs and proteins were measured by real-time PCR and immunohistochemistry (IHC). Mature activin-A, -B, -AB and follistatin proteins were measured by ELISA. Cancer tissues having ≤ the 20th percentile of the Smad4 IHC score were considered as low (L-S4) group. The Smad4-intact SW480 and Smad4-null HT29 colon cancer cell lines were treated with activins and follistatin, and cell cycle was analysed by flow cytometry. The cell cycle inducing (CCND1/CCND3) and inhibitory (p21/p27) proteins alongside the survival (survivin/BCL2) and pro-apoptosis (Casp-8/Casp-3) markers were measured by immunofluorescence. Thirty-nine patients had right-sided cancers (30%) and showed higher rates of L-S4 tumours (*n* = 17; 13.1%) alongside worse clinicopathological characteristics relative to left-sided cancers. The βA-subunit and activin-A increased, whilst βB-subunit and activin-AB decreased, in malignant sites and the late-stage cancers revealed the greatest abnormalities. Interestingly, follistatin declined markedly in early-stage malignant tissues, whilst increased significantly in the advanced stages. All activin molecules were comparable between the early stage right- and left-sided tumours, whereas the late-stage right-sided cancers and L-S4 tumours showed more profound deregulations. *In vitro*, activin-A increased the numbers of the SW480 cells in sub-G1 and G0/G1-phases, whereas reduced the HT29 cell numbers in the sub-G1 phase with simultaneous increases in the G0/G1 and S phases. The p21/p27/Casp-8/Casp-3 proteins escalated, whilst CCND1/CCND3/BCL2/survivin declined in the SW480 cells following activin-A, whereas activin-A only promoted p21 and p27 alongside reduced CCND3 in the HT29 cells. By contrast, activin-AB increased the numbers of SW480 and HT29 cells in Sub-G1 and G0/G1-phases and promoted the anti-cancer and reduced the oncogenic proteins in both cell lines. In conclusion, activins and follistatin displayed stage-dependent dysregulations and were markedly altered during the advanced stages of right-sided and L-S4 cancers. Moreover, the activin-A actions in CRC could be Smad4-dependent, whereas activin-AB may act as a Smad4-independent tumour suppressor protein.

## Introduction

Colorectal cancer (CRC) is the third most common malignancy and the fourth in cancer related deaths worldwide [1]. CRC is a heterogenous group of malignancies with divergent pathogenic molecular features that are, in part, dependent on tumour sidedness [2]. Primary tumour anatomical location is also an important prognostic factor and right-sided cancers (RSC) are frequently linked with mucinous histology, high-grade differentiation, poor prognosis, and worse survival rates [3, 4]. Furthermore, Smad4 is a tumour suppressor protein and malignant enterocytes commonly develop inactivating mutations in its gene during the late CRC stages, and loss of Smad4 protein has been linked with RSC and poor prognosis [5, 6].

Activins are plethoric proteins that belong to the transforming growth factor (TGF)-β family and normally mediate their cellular actions via Smad4 [7, 8]. The hetero- and homodimerization of two β-subunits (βA and βB) produce three mature proteins known as activin-A (βA-βA; Act-A), activin-B (βB-βB; Act-B) and activin-AB (βA-βB; Act-AB) [7, 8]. The biological actions of activins are tightly regulated by follistatin (FS), which equally neutralises all activin isoforms [9]. Activins initiate their signals by binding to their type II receptors that subsequently recruit activin type I receptors, also known as activin receptor-like kinases (ALKs), to activate the same intracellular mediators of TGF-β, Smads 2/3/4 [10]. While Act-A exclusively activates Smad4 by ALK4, both Act-B and Act-AB can trigger Smad4-dependent activities through ALK4 and ALK7 receptors [11, 12].

Act-A is the most studied isoform in CRC and the findings of several studies have demonstrated paradoxical actions for the protein, a phenomenon known as the “TGF-β paradox” or “molecular switch” [10, 13]. In this regard, a significant increase in Act-A has been observed during the early stages of cancer, suggesting a protective function against cancer progression by inducing cell cycle arrest and apoptosis [14-16]. Conversely, others have also reported an association between tumour progression and facilitation of cancer metastasis following an increase in Act-A levels at the late stages of malignancy [17-20]. Proposed explanations for this paradox involve the development of mutations by cancerous cells in the Smad4 gene to escape the growth inhibitory effects of Act-A [14, 15].

The available reports regarding the roles of Act-B, Act-AB, and FS in colon carcinogenesis are scarce [7, 21]. Furthermore, none of the previous studies investigated the expression of activins and FS in relation to primary tumour sidedness and Smad4 status. Hence, this study measured the genes and proteins of activins, FS and Smad4 in paired normal and cancerous colonic specimens and the results were analysed according to clinical stages, primary tumour sidedness, and Smad4 expression. The clinical findings were then confirmed by investigating the actions of the activins and FS on cell cycle progression and apoptosis markers in the Smad4-intact SW480 and Smad4-mutated HT29 colon cancer cells.

## Materials and Methods

### Clinical Studies

#### Sample Collection and Processing

Ethical approval was granted from the Institutional Review Boards of King Faisal Specialist Hospital and Research Centre (KFSH&RC; #RC-J/448/39) in Jeddah and King Abdullah Medical City (KAMC; #19-498) in Makkah prior to recruitment. Paired malignant and normal specimens were collected from two sources, namely archived Formalin-Fixed Paraffin-Embedded (FFPE), alongside another set of fresh tumours with their corresponding adjacent normal tissues following surgical resection. All patients from the FFPE and fresh tissue cohorts were Saudi citizens diagnosed with primary sporadic CRC and did not receive neoadjuvant chemo/radiotherapy before their curative/palliative surgery. Cases with inherited or recurrent CRC were excluded. The FFPE cohort of malignant, and their corresponding normal tissues, were obtained from 90 patients who had their surgery between January 2016 and June 2018. The tissue blocks were retrieved from the archives of the histopathology departments of both study sites following examination by expert histopathologists to assure adequacy.

The fresh specimens were collected from 40 patients between July 2018 and March 2020. Excised colon tissue was immediately transferred to the histopathology department and intratumoral, together with adjacent non-cancerous tissue specimens located at least 10 cm away from the tumour margins, were cut by an expert histopathologist after gross examination. The specimens were instantly submerged in RNA*Later* (#AM7021; Thermo Fisher Scientific; CA, United States) and stored at −80°C. The remaining surgical tissues were then fixed in 10% buffered formalin for 24 h and later processed by conventional histological techniques for pathological procedures, as mandated by routine institutional guidelines.

The final diagnosis and the histopathological staging were conducted by a consultant histopathologist according to the criteria of the 8th edition of the American Joint Committee on Cancer tumour-node-metastasis (TNM) staging system. As per the pathology and surgical reports, FFPE and fresh tumours located from the cecum to the proximal margin of splenic flexure were classified as RSC, whereas tumours located from the splenic flexure to the rectum were considered left-sided cancer (LSC).

#### Immunohistochemistry

All the primary antibodies were from Thermo Fisher Scientific. While rabbit polyclonal IgG antibodies were used to localise Smad4 (#PA5-34806), βB-subunit (#PA5-50818) and FS (#PA5-114319), the βA-subunit was detected by goat polyclonal IgG antibodies (#PA5-47004). Tissue sections (5 μm) were dewaxed in xylene, emersed in graded ethanol series, and then washed with deionized water. An avidin-biotin horseradish peroxidase technique was applied, and endogenous peroxidases were blocked with BLOXALL^®^ Endogenous Blocking Solution (#SP-6000-100; Vector Laboratories Inc., CA, United States). The slides were then washed in PBS for 10 min and normal goat (for rabbit 1^ry^ antibodies), or rabbit (for goat 1^ry^ antibodies) serum provided with the VECTASTAIN^®^ rabbit (#PK-6101) or goat (#PK-6105) Elite ABC-HRP Kits (Vector Laboratories Inc.) were then added to the sections for 30 min. The primary antibodies were subsequently added (1:150 concentration for all) followed by overnight incubation at 4°C. In the next day, the provided rabbit anti-goat or goat anti-rabbit biotinylated secondary antibodies alongside the avidin/biotin complex in the VECTASTAIN^®^ rabbit or goat Elite ABC-HRP Kits were prepared and used as per the manufacturer’s instructions. The negative control sections were treated similar to all other slides, but the primary antibodies were replaced with their equivalent isotype rabbit (#sc-2027) or goat (#sc-2028) IgG antibodies (Santa-Cruz Biotechnology; CA, United States). The sections were observed on a Leica DMi8 microscope (Leica Microsystems, Wetzlar, Germany) and Red/Green/Blue (RGB) digital images were captured from 10 non-overlapping fields with a ×20 objective.

The protein expression in the captured images was digitally analysed by ImageJ software (https://imagej.nih.gov/ij/) using the IHC Image Analysis Toolbox as described earlier [22, 23]. Briefly, the chromogen-stained areas were identified and isolated by the IHC tool and were assigned as the region of interest (ROI). To calculate the stain intensity, unstained/white areas in the RGB images had the maximum value of 255, whilst the IHC stained ROI were darker and generated values <255, thus caused an inverse relation between stain intensity and their scores. Hence, a reciprocal intensity scoring model was used to create positive associations between the IHC stain intensity and their scores as per the previously described equation [23]:

IHC stain intensity =[(255 − ROI stain score) × % ROI (ROI pixels /total image pixels × 100)]

We first analysed the intensity of Smad4 protein, and the percentiles (10th, 20th, 30th, etc.) of the reciprocal IHC scores in the cancer tissues were determined by SPSS software. Subsequently, the malignant samples with > the 20th percentile value of the Smad4 total IHC score were considered normal (N-S4), whilst those ≤ the 20th percentile value were classified as low Smad4 (L-S4) groups. The IHC scores for the remaining proteins were then compared between the paired normal and cancerous tissues of each patient, the clinical stages (early vs. advanced) and tumour sidedness (RSC vs. LSC) as well as between the N-S4 and L-S4 groups.

#### Enzyme Linked Immunosorbent Assay

Fresh normal and neoplastic colonic tissues weighing 0.5 g each were placed in 1 ml RIPA lysis buffer (#89900) with protease inhibitors (#78429; Thermo Fisher Scientific). Following centrifugation, the total protein concentrations were measured in the supernatants by a BCA Protein Assay (#A53225; Thermo Fisher Scientific). The protein samples were diluted with ultrapure deionized water for the final concentrations of 500 µg/ml. Act-A (#SEA001Hu), Act-B (#SEA170Hu), Act-AB (#SEA158Hu), and FS (#SEA391Hu) concentrations were then measured by ELISA using human specific kits (Cloud-Clone Corp.; TX, United States). All samples were processed in duplicate on a fully automated ELISA system (Human Diagnostics; Wiesbaden, Germany). The tissue concentrations of each protein were compared between the normal and cancerous tissues of each patient as well as between the cancer TNM stages (I/II vs. III/IV), tumour anatomical locations (RSC vs. LSC), and Smad4 status (N-S4 vs. L-S4).

#### Total RNA Extraction and Quantitative Reverse Transcription Polymerase Chain Reaction

All reagents were from Thermo Fisher Scientific. Total RNA was extracted by the PureLink™ RNA Mini Kit (#12183025) for fresh tissues and PureLink™ FFPE RNA Isolation Kit (#K156002) for archived specimens. The RNA quality and quantities were assessed by Qubit4 Fluorometer (Thermo Fisher Scientific).

The cDNA was synthesised from 1 μg of total RNA by a SuperScript™ VILO™ cDNA Synthesis Kit (#11754250). Biological replicates PCR reactions were performed in triplicate wells on an ABI^®^ 7500 system using the power SYBR Green master mix. Each well had 10 µl SYBR Green, 7 µl DNase/RNase free water, 1 µl of each primer (5 pmol; Supplementary Table S1) and cDNA (25 ng), and 40 amplification cycles (95°C/15 s and 60°C/1 min) were done. Negative controls included a minus-reverse transcription control and a minus-template PCR, in which nuclease free water was used as a template. Human *β-actin* gene was used to normalise the Ct values. The relative quantification of *IHNBA*, *INHBB*, *FST,* and *Smad4* genes in the malignant sites compared to their corresponding normal tissues was done by the 2^−∆∆Ct^ method.

### Cell Culture and Treatment

The Smad4-intact SW480 [24] and Smad4-mutated HT29 [25] colon cancer cell lines were acquired from the American Type Tissue Collection (ATCC; VA, United States). Biologically active Act-A (#338-AC-050/CF), Act-B (#659-AB-005/CF), Act-AB (#1066-AB-005/CF), and FS (#669-FO-025/CF) were obtained from R&D Systems (MN, United States). SW480 and HT29 cells were maintained in Dulbecco’s Modified Eagle’s Medium (DMEM; #31966047; Thermo Fisher) and Roswell Park Memorial Institute 1640 media (RPMI; #61870044; Thermo Fisher), respectively; both media were supplemented with 10% Foetal Bovine serum (FBS; #A3160802; Thermo Fisher) and 1% Antibiotic-Antimycotic solution (#15240062; Thermo Fisher), and the cells grown in a humified incubator at 37°C and 5% CO_2_.

For cell cycle analysis, 2 × 10^5^ SW480 and 3 × 10^5^ HT29 cells were seeded in 6-well plates for 24 h, whereas for immunofluorescence, cells were seeded in 8-well chamber slides (#229168; Celltreat™ Scientific Products; MA, United States) at 8,000 (SW480) and 16,000 (HT29) cells/well, also for 24 h. Concentrations (IC50) of Act-A (100 ng/ml), Act-AB (200 ng/ml) and FS (100 ng/ml) were determined using the 3-(4,5-Dimethylthiazol-2-yl)-2,5-Diphenyltetrazolium Bromide (MTT) cytotoxicity assay (data not shown). Act-B had little effect at the highest tested dose (500 ng/ml), and was therefore used at 200 ng/ml, matching the highest concentration of the activins, described above.

#### Cell Cycle Analysis

Following treatment with activins or follistatin, the cells were processed for cell cycle analysis according to Aslam et al. [26]. Briefly, cells were trypsinised, washed twice with PBS (500× g for 5 min), and then fixed in ice-cold 70% ethanol for 24 h at 4°C. After fixation, the cells were washed twice in PBS (600× g for 5 min) and then treated with 20 µg/ml RNase A (#12091021; Thermo Fisher) for 15 min, followed by the addition of 2 µg/ml propidium iodide (#P1304MP; Thermo Fisher). The stained cells were immediately analysed for cell cycle staging using a Novocyte 3000 flow cytometer (Agilent Technologies, CA, United States). The percentage of cells in the different stages (Sub-G1, G0/G1, S, G2/M) were determined for 20,000 acquired events using the NovoExpress software cell cycle algorithm, and data represents mean ± SD (*n* = 3).

#### Immunofluorescence Staining

Following activins or follistatin treatments for 24 h, the cells in the 8-well chamber slides were washed and then fixed for 15 min with 4% paraformaldehyde (#sc-281692; Santa-Cruz Biotechnology Inc.). The cells were then washed, permeabilised with 0.25% Triton X100 (#T8787; Sigma-Aldrich Co.; MO, United States) for 20 min, washed twice with PBS, and blocked for 30 min with normal donkey serum (#sc-2044; Santa-Cruz Biotechnology Inc.). Untreated SW480 and HT29 cells were also processed to confirm Smad4 status using 1:150 concentration of polyclonal rabbit IgG antibodies (#PA5-34806; Thermo Fisher).

The co-expression of cyclin D1 (CCND1) with cyclin-dependent kinase inhibitor-1B (p27), cyclin D3 (CCND3) with cyclin-dependent kinase inhibitor-1A (p21), survivin with caspase-8 (Casp-8), and B-cell lymphoma 2 (BCL2) with cleaved Casp-3 was examined in duplicate wells. The primary antibodies against p21 (#2947), p27 (#3686), survivin (#2808) and cleaved casp-3 (#9661) were monoclonal rabbit IgG antibodies (Cell Signaling Technology Inc., MA, United States), whereas CCND1 (#sc-8396), CCND3 (#sc-6283), BCL2 (#sc-7382) and Casp-8 (#sc-56070) were mouse monoclonal IgG antibodies (Santa-Cruz Biotechnology Inc.). Each set of primary mouse and rabbit antibodies was added to two wells/slide (1:200 concentration for all antibodies) and the slides were incubated for 3 h at room temperature. Following washing, the cells were incubated for 60 min with a mixture of tagged highly cross-adsorbed secondary donkey anti-rabbit (#A-31572; Alexa Fluor 555) and anti-mouse (#A-21202; Alexa Fluor 488) IgG antibodies (Thermo Fisher Scientific). The cells were counterstained with 4′,6-diamidino-2-phenylindole (DAPI; #D3571; Thermo Fisher Scientific), the detachable plastic wells were then removed, and the slides were cover-slipped with a permanent fluorescence mounting medium (#S3023; Dako, CA, United States).

All wells were observed with a Leica DMi8 microscope and digital images were captured within the same session from 10 random non-overlapping fields/well using a ×40 objective. The IF staining intensities of each targeted protein were measured by digital image analysis using the ImageJ software and are expressed as arbitrary units/cell numbers in analysed images, as previously described [27, 28].

### Statistical Analysis

All variables were assessed for normality by the Kolmogorov and Smirnov’s test and homogeneity by Levene test using SPSS version 25. Ordinal and discontinuous data are presented as numbers and percentages, and cross-tabulation followed by Chi-square (χ^2^) test were used for frequency analysis. Independent Student’s t or Mann-Whitney U tests were used to compare between two groups based on normality. Continuous data are shown either as mean ± standard deviation (SD) or median with interquartile range (IQR; 25th–75th percentiles), depending on data normality. Correlations were determined by Pearson’s or Spearman’s tests according to data normality. *p* value <0.05 was considered significant.

## Results

### Overall Clinicopathological Characteristics of the Fresh and FFPE Sample Cohorts

The FFPE (*n* = 90) and fresh (*n* = 40) tissue cohorts comprised 69 males (53.1%) and 61 females (46.9%), with average age comparable between both genders (63.3 ± 11.3 and 61.2 ± 12.7 years, respectively). The tumours were staged as T1 in six (4.6%), T2 in 21 (16.2%), T3 in 64 (49.5%) and T4 in 39 (30%) patients. Metastasis in regional lymph nodes was detected in 57 (43.9%) cases, whereas 18 patients (13.8%) had distant metastasis (stage M1a) in liver. Adenocarcinoma was the most common histology type (*n* = 108; 83.1%) and the remainders were mucinous carcinomas. Moreover, 15 (11.5%), 91 (70%) and 24 (18.5%) cases were diagnosed with poorly, moderately, and well-differentiated tumours, respectively. As per the TNM staging system, 60 (46.2%) patients were clinically classified as early (stages I/II) and the remainder (53.8%) as advanced (stages III/IV) cancers. Furthermore, the clinico-pathological features of tumours were similar between the fresh and FFPE sets (Table 1).

**TABLE 1:** The clinicopathological characteristics according to sample source.

	Fresh samples (*n* = 40; 30.8%)	FFPE samples (*n* = 90; 69.2%)	*p* Value
Mean ± SD of Age (year)	62.1 ± 11.9	62.8 ± 12.2	0.8
Gender			
Male	19 (14.6%)	50 (38.4%)	0.4
Female	21 (16.2%)	40 (30.8%)	
Tumour sidedness			
Right-sided	13 (10%)	26 (20%)	0.4
Left-sided	27 (20.8%)	64 (49.2%)	
Tumour infiltration (T stage)			
T1	2 (1.6%)	4 (3.1%)	0.9
T2	7 (5.4%)	14 (10.8%)	
T3	19 (14.6%)	45 (34.6%)	
T4	12 (9.2%)	27 (20.7%)	
Median (IQR) of tumour volume (cm^3^)	21.2 (9.6–49.5)	18 (8.9–39.1)	0.08
Regional lymph node (N stage)			
N0	18 (13.9%)	55 (42.3%)	0.1
N1	15 (11.5%)	19 (14.6%)	
N2	7 (5.4%)	16 (12.3%)	
Distant metastasis (M stage)			
M0	35 (26.9%)	77 (59.2%)	0.7
M1a	5 (3.9%)	13 (10%)	
Histology			
Adenocarcinoma	35 (26.9%)	73 (56.1%)	0.4
Mucinous	5 (3.9%)	17 (13.1%)	
Differentiation			
Poor	4 (3.1%)	11 (8.4%)	0.9
Moderate	28 (21.5%)	63 (48.5%)	
Well	8 (6.2%)	16 (12.3%)	
AJCC TNM stages			
Stages I/II (early)	15 (11.5%)	45 (34.6%)	0.2
Stages III/IV (advanced)	25 (19.3%)	45 (34.6%)	

Right-sided tumours were less prevalent (*n* = 39; 30%) in the FFPE and fresh samples and had markedly larger sizes alongside higher rates of advanced T stages (T3 and T4), distant metastasis, mucinous carcinoma, and poorly differentiated histology than the LSC group (Table 2). However, there was no significant difference between the RSC and LSC groups in relation to age, gender, N stages and the rates of early (I/II) and advanced (III/IV) cancer stages (Table 2).

**TABLE 2:** The clinicopathological characteristics according to tumour side. Bold values indicate statistical significance.

	Right-sided tumours (*n* = 39; 30%)	Left-sided tumours (*n* = 91; 70%)	*p* Value
Sample source			
Archived samples (FFPE)	26 (20%)	64 (49.2%)	0.7
Fresh samples	13 (10%)	27 (20.8%)	
Mean ± SD of Age (year)	64.1 ± 13.1	61.6 ± 11.4	0.3
Gender			
Male	23 (17.7%)	46 (35.4%)	0.4
Female	16 (12.3%)	45 (34.6%)	
Tumour infiltration (T stage)			
T1	0 (0%)	6 (4.6%)	**<0.001**
T2	0 (0%)	21 (16.1%)	
T3	20 (15.4%)	44 (33.9%)	
T4	19 (14.6%)	20 (15.4%)	
Median (IQR) of tumour volume (cm^3^)	29 (15–54)	12 (7–25)	**<0.0001**
Regional lymph node (N stage)			
N0	24 (18.4%)	49 (37.7%)	0.4
N1	7 (5.4%)	27 (20.8%)	
N2	8 (6.2%)	15 (11.5%)	
Distant metastasis (M stage)			
M0	25 (19.3%)	87 (66.9%)	**<0.0001**
M1a	14 (10.7%)	4 (3.1%)	
Histology			
Adenocarcinoma	26 (20%)	82 (63.1%)	**0.001**
Mucinous	13 (10%)	9 (6.9%)	
Differentiation			
Poor	11 (8.5%)	4 (3.1%)	**<0.0001**
Moderate	27 (20.8%)	64 (49.2%)	
Well	1 (0.7%)	23 (17.7%)	
AJCC TNM stages			
Stages I/II (early)	13 (10%)	47 (36.1%)	0.06
Stages III/IV (advanced)	26 (20%)	44 (33.9%)	

### Clinicopathological Characteristics According to Smad4 Status

The Smad4 mRNA was markedly decreased in the early-stage (3.2-fold) and advanced-stage (11.1-fold) neoplastic tissues relative to their corresponding normal specimens (*p* < 0.001 for both), and the levels were lowest in the late-stage malignant tissues.

**FIGURE 1:**
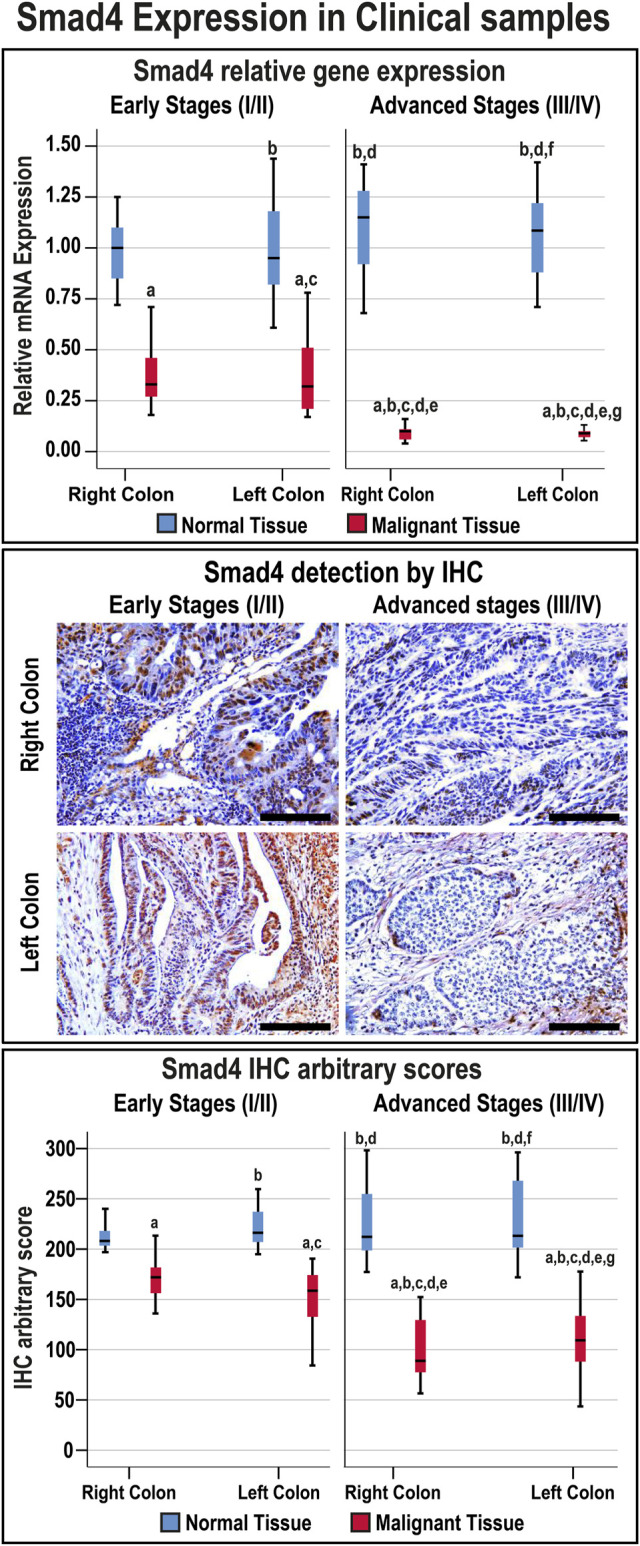
Relative expression (median) of Smad4 gene and protein in addition to the protein localisation by IHC in the FFPE (*n* = 90 patients) and fresh (*n* = 40 patients) cohorts of paired malignant and normal colonic tissues according to clinical stages and tumour sidedness (Scale bar = 15 μm; a = *p* < 0.05 compared with right-sided normal tissue from early cancer; b = *p* < 0.05 compared with early stage right-sided colon cancer; c = *p* < 0.05 compared with left-sided normal tissue from early cancer; d = *p* < 0.05 compared with early stage left-sided colon cancer; e = *p* < 0.05 compared with right-sided normal tissue from advanced cancer; f = *p* < 0.05 compared with advanced stage right-sided colon cancer and g = *p* < 0.05 compared with left-sided normal tissue from advanced cancer).

Smad4 protein was exclusively localised in the epithelial nuclei and the expression in normal tissues was comparable between the FFPE and fresh samples, anatomical sites, and cancer stages (Supplementary Figure S1). Nevertheless, Smad4 immunostain was markedly (1.4-fold and 1.9-fold, respectively) weaker in the tumorous tissues from early and advanced cancer stages compared with their corresponding normal specimens (Figure 1; *p* < 0.001). Smad4 protein in the malignant tissues from advanced stages was also significantly lower than the early cancer stages (*p* < 0.05). However, the expression of Smad4 was similar between the RSC and LSC during either the early or advanced stages of CRC (Figure 1).

The overall median of the Smad4 IHC scores in the FFPE and fresh malignant sites was 130.4 (96.2–157.64), and 26 patients (20%) showed IHC scores ≤ the 20th percentile cut-off value (87.44) and were classified as the L-S4 group. Moreover, the frequencies of low Smad4 protein were not significantly different between the FFPE and fresh malignant specimens, and the age was comparable between the N-S4 and L-S4 groups (Table 3). However, the LS-4 group was associated with markedly larger tumour sizes, advanced T and N stages, distant metastasis, mucinous carcinoma, poorly differentiated histology, and RSC than the N-S4 group. Moreover, the late-stage tumours (III/IV) were significantly more prevalent in the L-S4 group (Table 3).

**TABLE 3:** The clinicopathological characteristics according to Smad4 status in the FFPE and fresh malignant tissues (*n* = 130). Bold values indicate statistical significance.

	Normal Smad4 (*n* = 104; 80%)	Low Smad4 (*n* = 26; 20%)	*p* Value
Sample source			
Archived samples (FFPE)	71 (54.6%)	19 (14.6%)	0.6
Fresh samples	33 (25.4%)	7 (5.4%)	
Mean ± SD of Age (year)	62.3 ± 11.9	62.7 ± 12.1	0.8
Gender			
Male	58 (46.6%)	11 (8.5%)	0.2
Female	46 (35.4%)	15 (11.5%)	
Tumour anatomical site			
Right-sided tumour	22 (16.9%)	17 (13.1%)	**<0.0001**
Left-sided tumour	82 (63.1%)	9 (6.9%)	
Tumour infiltration (T stage)			
T1	5 (3.8%)	1 (0.7%)	**0.008**
T2	21 (16.2%)	0 (0%)	
T3	53 (40.8%)	11 (8.5%)	
T4	25 (19.2%)	14 (10.8%)	
Median (IQR) of tumour volume (cm^3^)	12.25 (8.5–25)	38.5 (19.5–55.75)	**<0.0001**
Regional lymph node (N stage)			
N0	60 (46.2%)	13 (10%)	**<0.0001**
N1	32 (24.6%)	2 (1.5%)	
N2	12 (9.2%)	11 (8.5%)	
Distant metastasis (M stage)			
M0	101 (77.7%)	11 (8.5%)	**<0.0001**
M1a	3 (2.3%)	15 (11.5%)	
Histology			
Adenocarcinoma	93 (71.5%)	15 (11.5%)	**<0.0001**
Mucinous	11 (8.5%)	11 (8.5%)	
Differentiation			
Poor	7 (5.4%)	8 (6.2%)	**0.002**
Moderate	75 (57.7%)	16 (12.3%)	
Well	22 (16.9%)	2 (1.5%)	
AJCC TNM stages			
Stages I/II (early)	59 (45.4%)	1 (0.7%)	**< 0.0001**
Stages III/IV (advanced)	45 (34.6%)	25 (19.3%)	

### Activin Subunits and Follistatin Gene Expression

**FIGURE 2:**
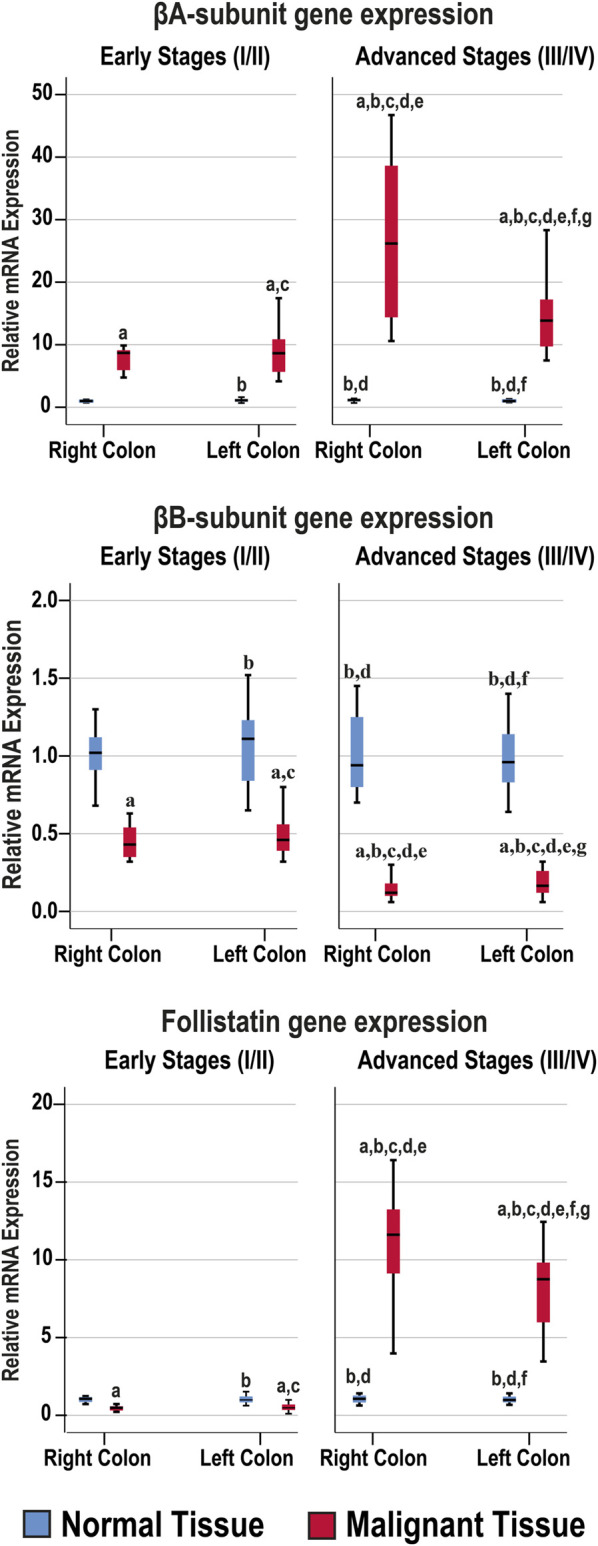
Relative expression (median) of activin β-subunits and follistatin mRNAs in the FFPE (*n* = 90 patients) and fresh (*n* = 40 patients) cohorts of paired malignant and normal colonic tissues according to clinical stages and tumour sidedness (a = *p* < 0.05 compared with right-sided normal tissue from early cancer; b = *p* < 0.05 compared with early stage right-sided colon cancer; c = *p* < 0.05 compared with left-sided normal tissue from early cancer; d = *p* < 0.05 compared with early stage left-sided colon cancer; e = *p* < 0.05 compared with right-sided normal tissue from advanced cancer; f = *p* < 0.05 compared with advanced stage right-sided colon cancer and g = *p* < 0.05 compared with left-sided normal tissue from advanced cancer).

The mRNA levels of the targeted genes in normal colonic tissues, from the FFPE and fresh cohorts of specimens, were similar between the RSC and LSC as well as between the early and late malignancy stages within each set of specimens (Figure 2). While the βA-subunit mRNA was significantly (8.5-fold) higher, the βB-subunit (2.3-fold) and FS (2.1-fold) decreased markedly in the neoplastic tissues of the early stages compared with their corresponding normal samples (*p* < 0.001 for all genes). Although the gene expression of β-subunits in the early-stage malignant tissues was equal between the RSC and LSC groups, the latter showed marked (21-fold) increases in the βA-subunit alongside (6.2-fold) declines in the βB-subunit genes in the late stages of CRC (Figure 2; *p* < 0.0001). Interestingly, the advanced-stage cancer specimens showed significant and paradoxical (9.6-fold) increases in FS mRNA, relative to their corresponding normal tissues, and were also substantially higher than the early cancer tissues (*p* < 0.0001). Moreover, the RSC samples from late stages showed the greatest rise in the βA-subunit and FS gene expression alongside the lowest βB-subunit mRNA levels compared with all other tissues (Figure 2).

The β-subunits and FS mRNAs were also equal between the normal specimens corresponding to the N-S4 and L-S4 cancerous tissues. However, the βA-subunit (34.9; IQR: 13.52–39.13) and FS (12.57; IQR: 8.35–14.88) mRNAs were markedly higher in the L-S4 malignant specimens relative to the N-S4 tumours (9.85; IQR: 7.64–14.35 and 0.78; IQR: 0.48–8.85, respectively; *p* < 0.0001). In contrast, the βB-subunit gene expression was significantly reduced in the malignant tissues with low Smad4 (0.16; IQR: 0.1–0.21) than the cancerous samples with normal Smad4 expression (*p* < 0.0001).

### Activin β-Subunits and Follistatin Protein Expression by IHC

**FIGURE 3:**
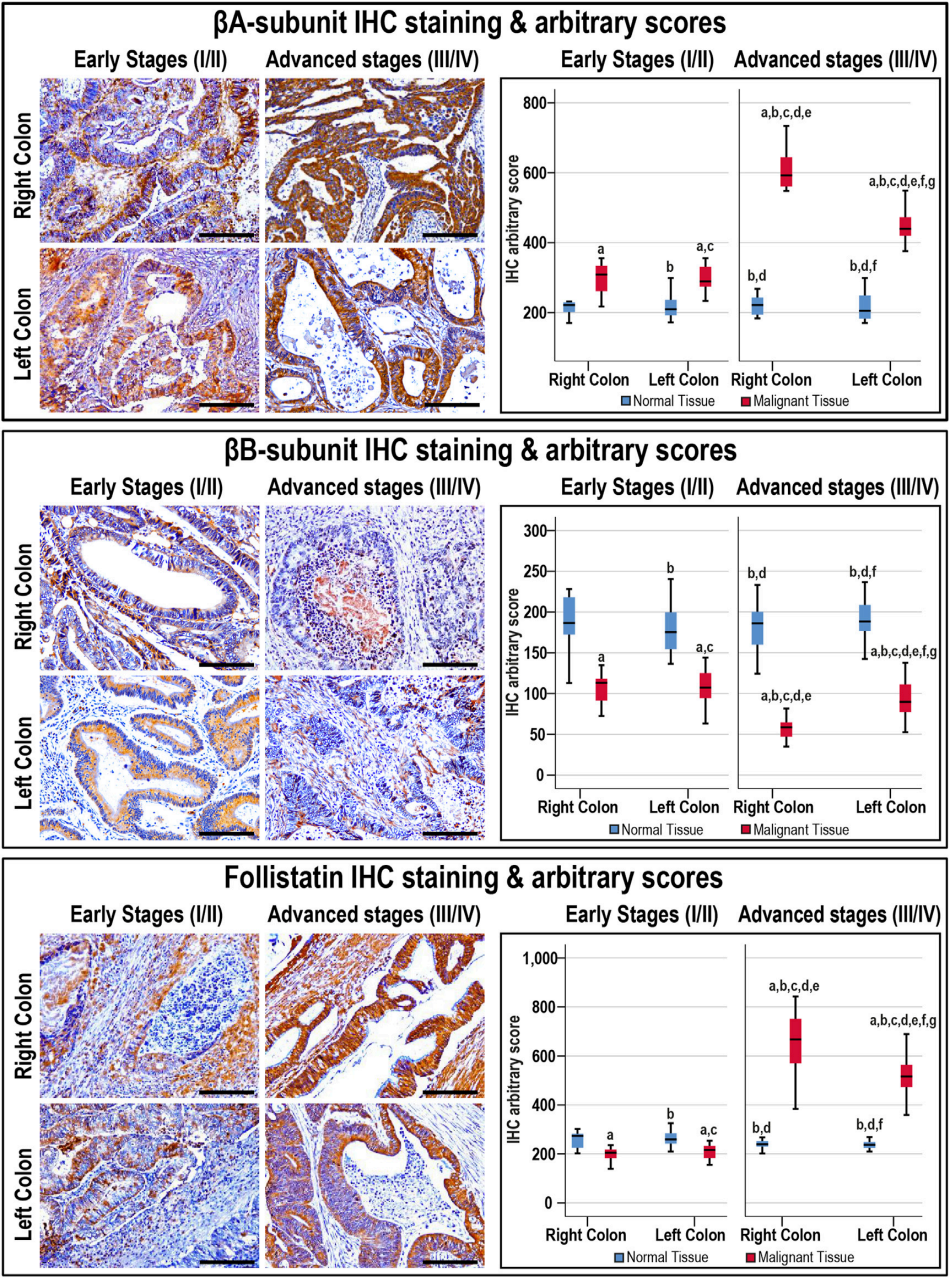
Localisation of activin βA- and βB-subunits alongside follistatin proteins by immunohistochemistry (IHC) in the FFPE (*n* = 90 patients) and fresh (*n* = 40 patients) cohorts of malignant colonic tissues according to clinical stages and tumour sidedness (Scale bar = 15 μm). Additionally, the relative protein expression of the targeted molecules in the malignant and normal colonic tissues according to cancer stage and tissue sidedness is shown as boxplots (a = *p* < 0.05 compared with right-sided normal tissue from early cancer; b = *p* < 0.05 compared with early stage right-sided colon cancer; c = *p* < 0.05 compared with left-sided normal tissue from early cancer; d = *p* < 0.05 compared with early stage left-sided colon cancer; e = *p* < 0.05 compared with right-sided normal tissue from advanced cancer; f = *p* < 0.05 compared with advanced stage right-sided colon cancer and g = *p* < 0.05 compared with left-sided normal tissue from advanced cancer).

The antibodies against the β-subunits and FS clearly labelled the cytoplasm of normal luminal and glandular colonic epithelia (Supplementary Figure S1). Moreover, there were no significant differences in the targeted proteins between the normal specimens according to sample sources, anatomical sites, and cancer stages (Figure 3).

In cancerous tissues, the βA-subunit was mainly localised in the cytoplasm of carcinomas with scarce stromal staining, and the early and late cancer stages showed markedly (1.4-fold and 2.3-fold, respectively) higher expression relative to their corresponding normal tissues (*p* < 0.001). Furthermore, the βA-subunit stain intensity in the malignant tissues was stronger in late than early stages of cancer (Figure 3; *p* < 0.01). In contrast, the βB-subunit (1.7-fold and 2.6-fold, respectively) diminished markedly in the tumorous tissues from early and advanced stages of CRC compared with their corresponding normal samples (*p* < 0.001). Moreover, the βB-subunit was almost undetectable in the late-stage cancer tissues and was substantially weaker than the early stages of malignancy (Figure 3; *p* < 0.01). Although FS (1.3-fold) declined significantly in the early stages of cancer, and was mostly located in the stroma surrounding tumours, the protein (2.5-fold) escalated markedly and showed strong cytoplasmic staining in advanced cancers than their corresponding normal tissues (Figure 3; *p* < 0.001). Follistatin IHC scores were also higher in the advanced than early stages of cancer (*p* < 0.0001). According to tumour sidedness, there were no significant differences in the expression of activin β-subunits and FS during the early stages of malignancy between the RSC and LSC groups. However, the RSC group showed (1.4-fold and 1.3-fold) higher βA-subunit and FS proteins alongside (1.7-fold) lower βB-subunit in the late cancer stages (Figure 3).

Moreover, the βB-subunit protein was equal in the normal specimens from the N-S4 (188.2; IQR: 166.8–210.8) and L-S4 (176.8; IQR: 159.8–187.6) groups. However, the βA-subunit (209.7; IQR: 185.9–237.1 vs. 219.8; IQR: 202.7–253, respectively; *p* = 0.03) and FS (248.4; IQR: 229.8–270.7 vs. 233.1; IQR: 226.4–254.2, respectively; *p* = 0.007) were higher in the normal tissues from the L-S4 than the N-S4 group. The L-S4 tumours also had higher βA-subunit (621.2; IQR: 433.9–701.6 vs. 340.2; IQR: 287.6–442.4) and FS (688.4; IQR: 479.9–773.3 vs. 237.4; IQR: 287.6–631) alongside lower βB-subunit (61.4; IQR: 42.7–82.3 vs. 99.03; IQR: 83.4–118.1) proteins relative to cancers with normal Smad4 (*p* < 0.0001 for all).

### Activins and Follistatin in Fresh Colonic Tissue Homogenates

**FIGURE 4:**
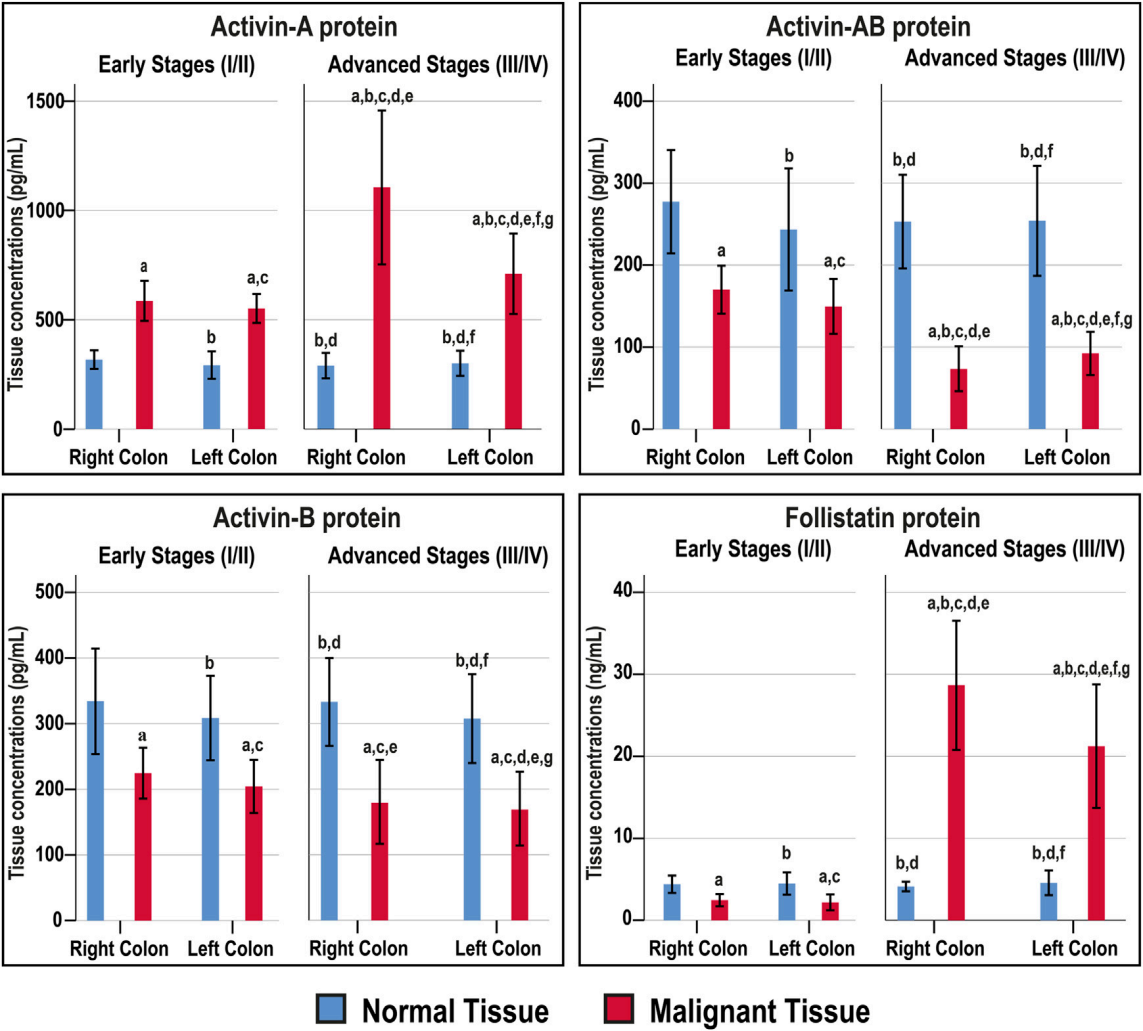
Concentrations (mean ± SD) of activin-A, activin-B, activin-AB and follistatin in the fresh cohort of paired malignant and normal colonic tissues (*n* = 40 patients) according to clinical stages and tumour sidedness (a = *p* < 0.05 compared with right-sided normal tissue from early cancer; b = *p* < 0.05 compared with early stage right-sided colon cancer; c = *p* < 0.05 compared with left-sided normal tissue from early cancer; d = *p* < 0.05 compared with early stage left-sided colon cancer; e = *p* < 0.05 compared with right-sided normal tissue from advanced cancer; f = *p* < 0.05 compared with advanced stage right-sided colon cancer and g = *p* < 0.05 compared with left-sided normal tissue from advanced cancer).

The Act-A, Act-AB, Act-B, and FS concentrations were similar between the right and left-sided normal specimens as well as between normal tissues obtained from the early and late stages of CRC (Figure 4). The intratumoral Act-A levels were markedly higher than their corresponding normal tissues, showed steady increases with cancer progression, and the highest levels were detected in the advanced malignancy stages from the RSC group (Figure 4). In contrast, both Act-AB and Act-B decreased markedly in the malignant specimens than their corresponding normal tissues. While Act-AB levels were significantly lower in the tumorous samples from late compared with early cancer stages, the lowest amounts were seen in the late-stage RSC specimens (Figure 4). Intratumoral Act-B, however, was equal between the early and late stages of cancer, as well as, between the RSC and LSC samples. Contrariwise, FS was markedly lower in the early-stage cancer tissues compared with their corresponding normal samples, whereas the levels surged drastically in late-stage tumours relative to the normal and early-stage cancer tissues, and the highest amounts were detected in the right-sided malignancies (Figure 4).

The normal tissues from the N-S4 and L-S4 had equal levels of Act-A (299.3; IQR: 260.7–333.1 vs. 291; IQR: 255.6–321 pg/ml), Act-AB (268.6; IQR: 187.5–304.5 vs. 268.2; IQR: 218.1–287.1 pg/ml), Act-B (304.6; IQR: 274–363.1 vs. 328.4; IQR: 277–363.1 pg/ml), and FS (4.2; IQR: 3.8–5.6 vs. 4; IQR: 3.6–4.8 ng/ml). In contrast, the L-S4 malignant tissues had markedly higher Act-A (1332.5; IQR: 791–1638.8 vs. 649.2; IQR: 535.9–794 pg/ml; *p* = 0.001) and FS (28.7; IQR: 24.6–32.1 vs. 14.4; IQR: 2.2–24.4 ng/ml, *p* < 0.01) than the N-S4 cancerous specimens. By contrast, the neoplastic tissue Act-B amounts (119.8; IQR: 75.1–197 vs. 201.4; IQR: 144.5–233 pg/ml, respectively; *p* < 0.01) and Act-AB (62.1; IQR: 53.1–96.7 vs. 123.8; IQR: 75.9–153.7 pg/ml; *p* < 0.001) were substantially lower in the L-S4 group.

Act-A and FS intratumoral levels correlated together directly (r = 0.722; *p* < 0.0001), whereas they showed inverse associations with Act-AB levels (r = −0.728 and r = −0.641, respectively; *p* < 0.0001). However, Act-B levels in cancerous tissues did not correlate with Act-A (r = −0.249), Act-AB (r = 0.087), and FS (r = −0.027). Moreover, Act-A and FS in malignant tissues linked positively, whereas Act-AB associated negatively, with tumour size, sidedness, histology, tumour grade, numbers of positive lymph nodes, TNM stages, and Smad4 status (Table 4).

**TABLE 4:** Spearman’s Correlations between the clinicopathological characteristics of fresh colorectal cancer samples (*n* = 40) and intratumoral concentrations of mature activins and follistatin.

	Activin-A	Activin-B	Activin-AB	Follistatin
Age	0.119	0.024	0.072	−0.056
Gender	0.232	−0.071	−0.310	0.176
Right-sided cancer	0.321*	-0.011	0.243	0.331*
T stage	0.397*	0.229	−0.316*	0.515**
Tumour Size	0.645**	0.240	−0.564***	0.777***
N Stage	0.498**	0.217	−0.641***	0.708***
Numbers of positive lymph nodes	0.500**	0.216	−0.618***	0.727***
M Stage	0.534***	-0.380*	−0.409*	0.311
Mucinous Carcinoma	0.351*	0.131	−0.154	0.475**
Poor differentiation	0.425**	−0.270	−0.391*	0.369*
Low Smad4 expression	−0.499**	0.091	0.408**	−0.430**

*p < 0.05.

**p < 0.01.

***p < 0.001.

### Effects of Activins and Follistatin on Cell Cycle Progression in the SW480 and HT29 Cell Lines

**FIGURE 5:**
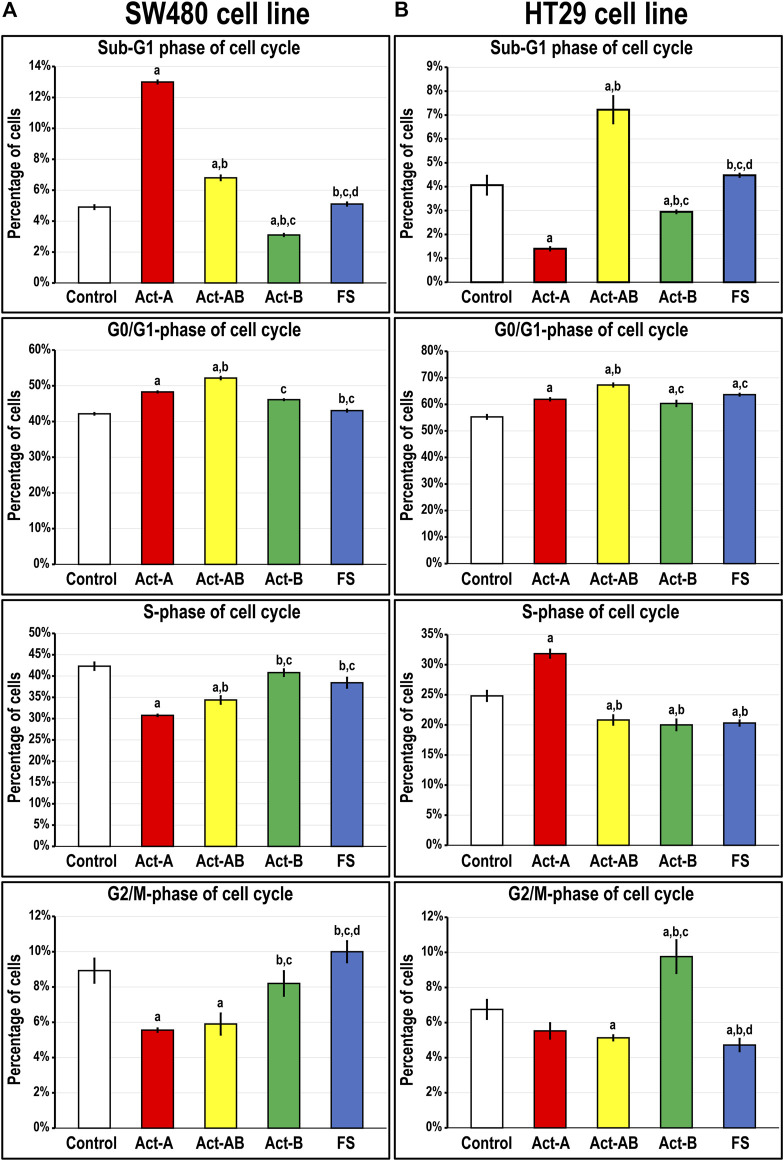
Percentages of cells (mean ± SD) in the different phases of cell cycle in untreated control cells, and following treatments with Act-A, Act-AB, Act-B or FS for 24 h in the **(A)** Smad4-intact SW480 and **(B)** Smad4-negative HT29 cell lines (a = *p* < 0.05 compared with control untreated cells; b = *p* < 0.05 compared with Act-A; c = *p* < 0.05 compared Act-AB and d = *p* < 0.05 compared with Act-B).

Smad4 protein was detected in the untreated SW480 cells and showed perinuclear localisation, whereas it was negligible in the HT29 cells (Supplementary Figure S1). Act-A and Act-AB markedly increased the numbers of SW480 cells in the sub-G1 phase of cell cycle relative to untreated cells, where Act-A induced the highest numbers (Figure 5A). The percentages of cells in the G0/G1-phase also increased significantly, with declines in the S and G2/M phases, after treatment with the same two proteins relative to untreated controls cells. In contrast, the different cell cycle phases were unaffected by the addition of either Act-B or FS, compared to non-treated SW480 cells (Figure 5A).

On the other hand, Act-A significantly reduced the numbers of HT29 cells in the sub-G1 phase, whilst simultaneously increasing the percentages in the G0/G1 and S-phases, relative to control cells (Figure 5B). The Act-B effects on the HT29 cell numbers in the sub-G1 and G0/G1-phases mimicked those of Act-A but were much weaker. Moreover, Act-B increased the cell counts significantly in the G2/M-phase of cell cycle, not observed with Act-A treatment. Contrariwise, Act-AB showed the highest cell proportions in the Sub-G1 and G0/G1 phases, with concomitant declines in the S- and G2/M-phases, relative to control cells (Figure 5B). Although the HT29 cell numbers in the G0/G1-phase were also significantly higher following FS treatment compared with control, the numbers were significantly lower than Act-AB (Figure 5B). Moreover, FS markedly reduced the numbers of HT29 cells in the S and G2/M, but not sub-G1, phases of cell cycle relative to non-treated cells.

### Expression of Cell Cycle Regulatory Proteins in the SW480 and HT29 Cell Lines Following Activins and Follistatin Treatment

**FIGURE 6:**
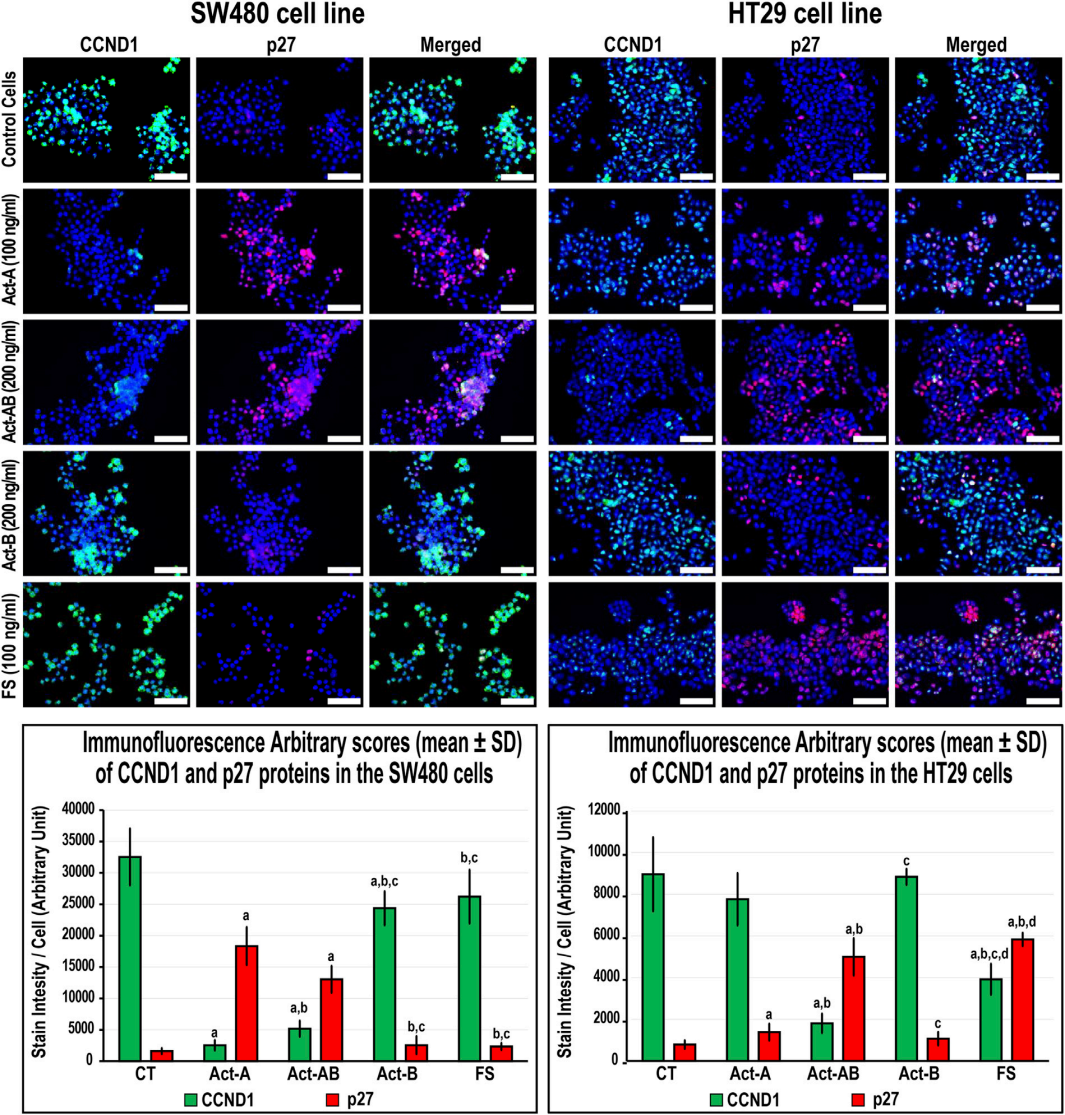
Co-expression of CCND1 (green) with p27 (red) proteins by immunofluorescence in untreated control cells and following 24 h treatment with Act-A (100 ng/ml), Act-AB (200 ng/ml), Act-B (200 ng/ml) or FS (100 ng/ml) in the SW480 and HT29 cell lines (Scale bar = 8 μm). In addition, the arbitrary scores of the immunofluorescent stain intensity/cell (mean ± SD) for each protein are shown as graph bars (a = *p* < 0.05 compared with control untreated cells; b = *p* < 0.05 compared with Act-A; c = *p* < 0.05 compared Act-AB and d = *p* < 0.05 compared with Act-B).

**FIGURE 7:**
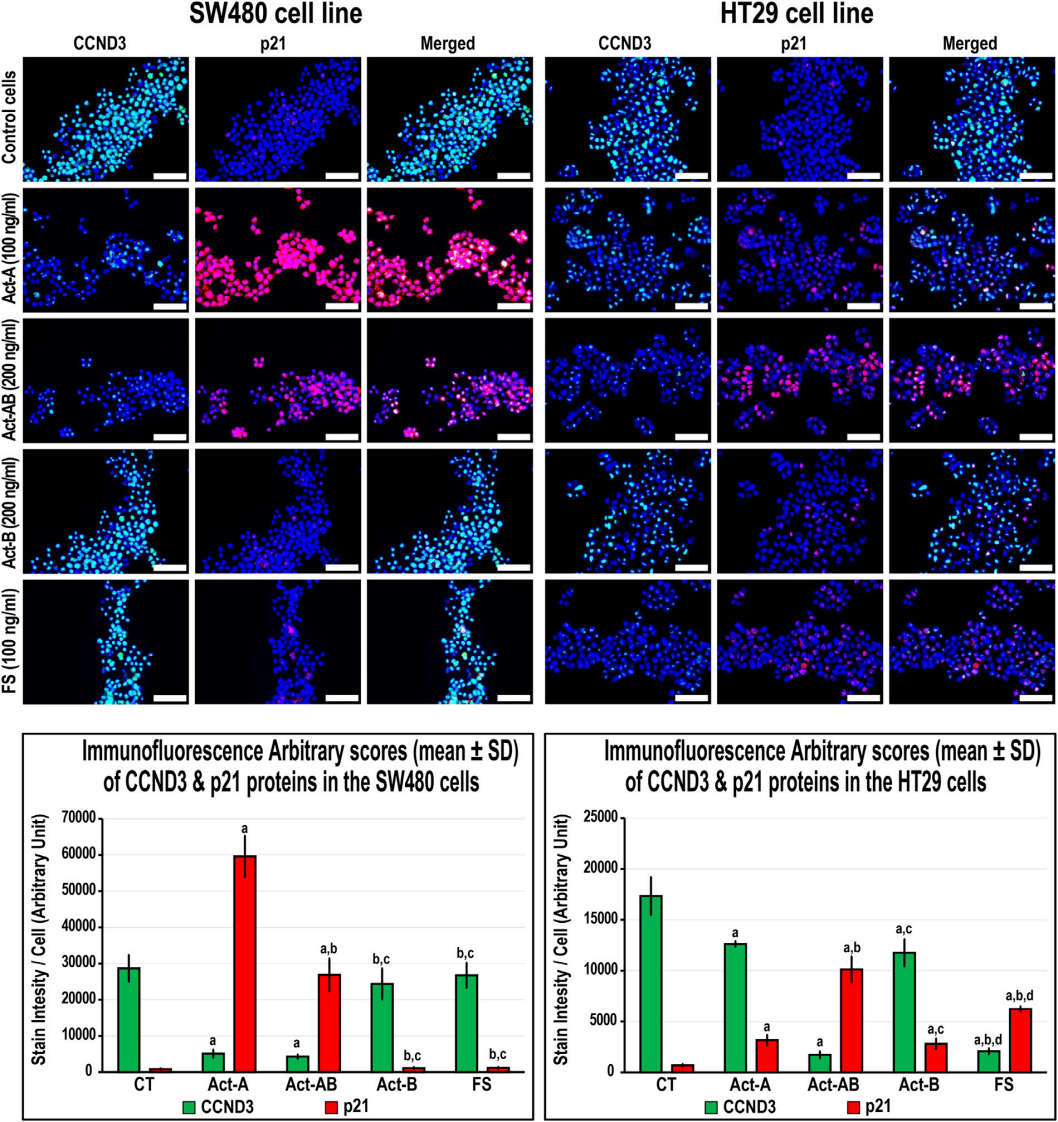
Co-expression of CCND3 (green) with p21 (red) proteins by immunofluorescence in untreated control cells and following 24 h treatment with Act-A (100 ng/ml), Act-AB (200 ng/ml), Act-B (200 ng/ml) or FS (100 ng/ml) in the SW480 and HT29 cell lines (Scale bar = 8 μm). In addition, the arbitrary scores of the immunofluorescent stain intensity/cell (mean ± SD) for each protein are shown as graph bars (a = *p* < 0.05 compared with control untreated cells; b = *p* < 0.05 compared with Act-A; c = *p* < 0.05 compared Act-AB and d = *p* < 0.05 compared with Act-B).

In the SW480 cells, CCND1 (Figure 6) and CCND3 (Figure 7) proteins declined markedly with Act-A and Act-AB treatments compared with control cells. Moreover, Act-A and Act-B increased the p27 and p21 proteins in SW480 cells relative to untreated cells (Figures 6, 7). Whilst Act-A and Act-AB also promoted the p27 and p21 protein expression alongside reduced CCND3 levels in the HT29 cells, the effects of Act-AB were significantly higher than the untreated and Act-A-treated cells. However, the expression of CCND1 in the HT29 cells only weakened significantly with Act-AB treatment (5-fold; Figure 6).

Interestingly, Act-B only diminished CCND1 in the SW480 cells (Figure 6), whilst enhanced the expression of p21 and lowered CCND3 in the HT29 cells (Figure 7); but its effects were weaker than the other activins. On the other hand, the cell cycle regulatory proteins were comparable between the FS-treated and untreated SW480 cells, whereas FS markedly increased p27 and p21 levels and concurrently reduced CCND3 and CCND1 proteins expression in the HT29 cells relative to control cells (Figures 6, 7).

### Expression of Proliferation and Apoptosis Proteins in the SW480 and HT29 Cell Lines Following Activins and Follistatin Treatments

**FIGURE 8:**
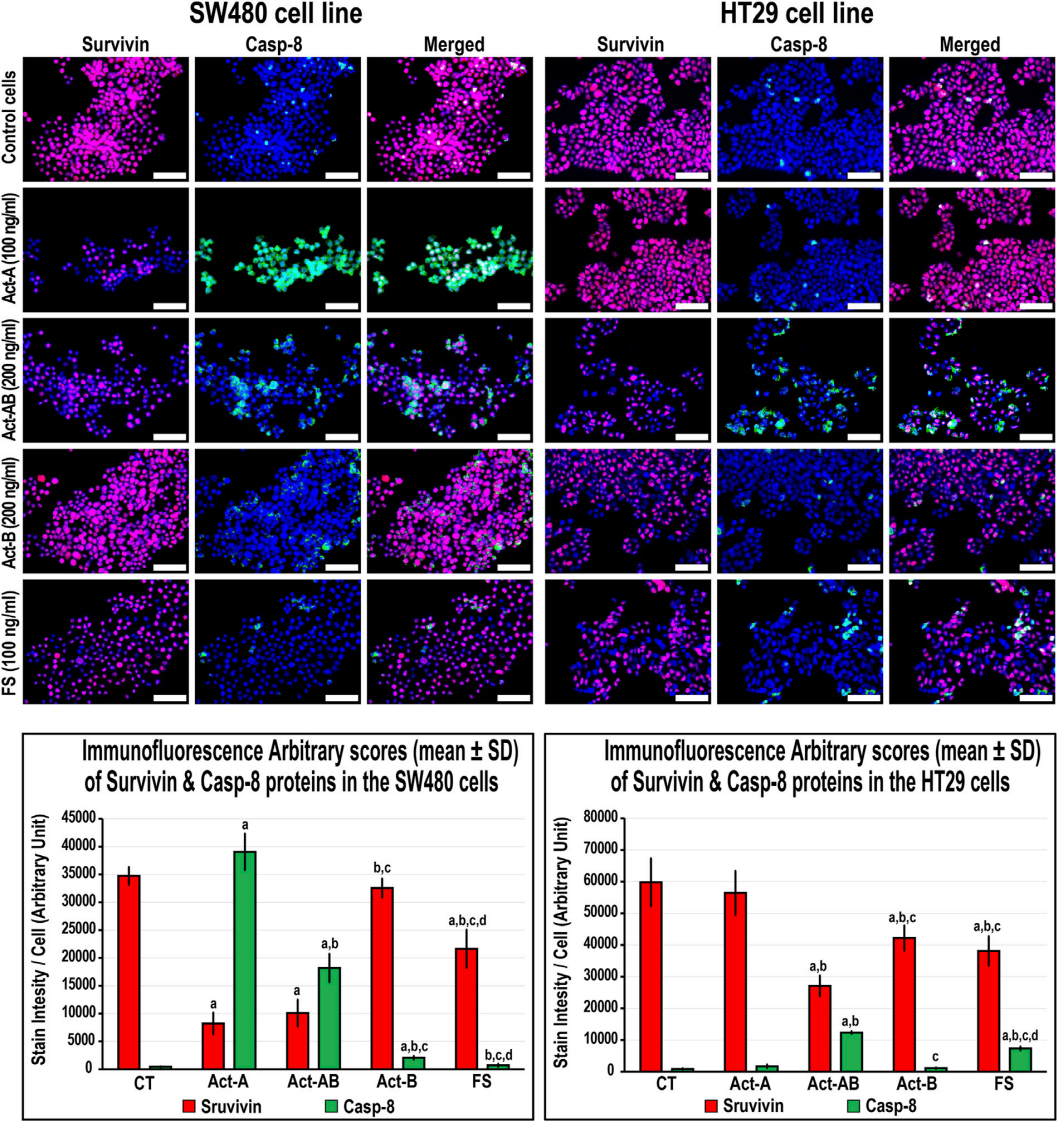
Co-expression of survivin (red) with Casp-8 (green) proteins by immunofluorescence in untreated control cells and following 24 h treatment with Act-A (100 ng/ml), Act-AB (200 ng/ml), Act-B (200 ng/ml) or FS (100 ng/ml) in the SW480 and HT29 cell lines (Scale bar = 8 μm). In addition, the arbitrary scores of the immunofluorescent stain intensity/cell (mean ± SD) for each protein are shown as graph bars (a = *p* < 0.05 compared with control untreated cells; b = *p* < 0.05 compared with Act-A; c = *p* < 0.05 compared Act-AB and d = *p* < 0.05 compared with Act-B).

**FIGURE 9:**
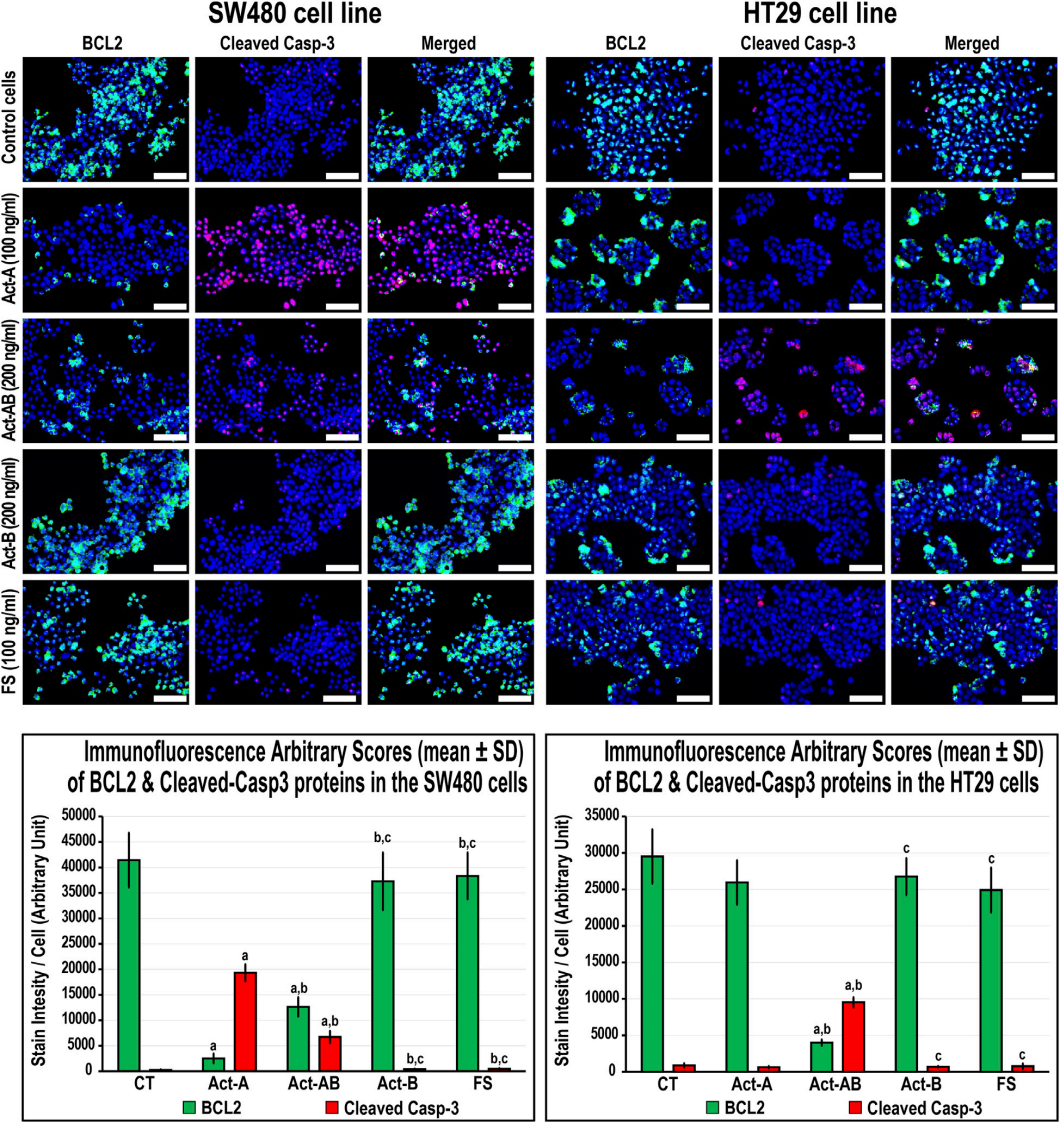
Co-expression of BCL2 (green) with cleaved Casp-3 (red) proteins by immunofluorescence in untreated control cells and following 24 h treatment with Act-A (100 ng/ml), Act-AB (200 ng/ml), Act-B (200 ng/ml) or FS (100 ng/ml) in the SW480 and HT29 cell lines (Scale bar = 8 μm). In addition, the arbitrary scores of the immunofluorescent stain intensity/cell (mean ± SD) for each protein are shown as graph bars (a = *p* < 0.05 compared with control untreated cells; b = *p* < 0.05 compared with Act-A; c = *p* < 0.05 compared Act-AB and d = *p* < 0.05 compared with Act-B).

The expression of survivin decreased, whereas Casp-8 increased, significantly in the SW480 cells with Act-A and Act-AB treatments compared with untreated cells (Figure 8). Likewise, Act-A and Act-AB lowered BCL2 and increased cleaved Casp-3 in the SW480 cells (Figure 9). However, Act-A showed no effect on the proliferation and apoptosis proteins in the HT29 cells, whereas Act-AB markedly lessened survivin and BCL2, alongside boosted Casp-8 and cleaved Casp-3 relative to control cells (Figures 8, 9).

Although Act-B in the SW480 cells only showed limited increases in Casp-8 protein relative to control, the expression of BCL2, survivin and Casp-3 were equal between both groups (Figures 8, 9). In the HT29 cells, Act-B only decreased survivin marginally and had no effect on BCL2, Casp-8, and Casp-3 proteins. In contrast, FS reduced survivin in both cell lines, whilst increased Casp-8 only in the HT29 cells. Moreover, the BCL2 and Casp-3 proteins were equal between the FS-treated and control cells in both cell lines (Figures 8, 9).

## Discussion

This study measured activins and follistatin in malignant colon tissues and the results were compared between the early and late clinical stages of cancer according to tumour sidedness and Smad4 expression. Additionally, the effects of activins and follistatin proteins related to cell cycle and expression of apoptosis markers were investigated in the Smad-4 intact SW480 and Smad-mutated HT29 colon cancer cells. Similar to earlier reports from Saudi Arabia [29, 30], the right-sided lesions were less frequent (30%) in our cohorts of samples and were associated with larger tumour sizes, more advanced T stage, poor differentiation, mucinous carcinoma, and distant metastasis, which agree with several previous studies [3, 4]. Our findings also showed marked decreases in the mRNA and protein of Smad4 in malignant tissues and the lowest levels were seen in the RSC group advanced stages. In agreement with this, loss-of-function mutations in Smad4 are common during late stages of CRC, especially with right-sided tumours, and loss of Smad4 has been linked with metastasis and worse prognosis [5, 6].

Our results also revealed progressive increases in the βA-subunit gene and protein together with Act-A concentrations in cancerous specimens relative to their corresponding normal samples, and the late-stage neoplastic tissues had markedly higher amounts than the early stages. Act-A levels in malignant samples also correlated directly with tumour sizes, TNM stages, counts of positive lymph nodes and distant metastasis. The RSC advanced stages and the L-S4 malignant tissues had substantially greater amounts of βA-subunit and Act-A. Moreover, Act-A treatment markedly boosted the numbers of the SW480 cells in sub-G1 and G0/G1-phases, increased the cell cycle inhibitory (p21/p27) and pro-apoptotic (Casp-8/Casp-3) proteins, and decreased the markers of cell cycle progression (CCND1/CCND3) and cell survival (BCL2/survivin). In contrast, Act-A significantly reduced the numbers of HT29 cells in the sub-G1 phase with concurrent increases in the G0/G1 and S phases. While Act-A modestly induced the p21 and p27 proteins in the HT29 cells, it had no effect on the cell proliferation and apoptosis markers.

Correlating with our data, Act-A triggered p21-dependent cell cycle arrest and apoptosis in Smad4-intact colon cancer cells, whereas the deletion of *Smad4* gene attenuated the protein anti-cancer activities [31, 32]. Concurrently, restoring activin type IIA receptor in mutated malignant enterocytes provoked smad4-dependent cell cycle arrest and apoptosis, implying that cancerous cells could bypass the Act-A anti-cancer actions by developing mutations in the *Acvr2a* or *Smad4* genes [14, 15]. Moreover, CRC induction in murine elevated Act-A and inhibited Smad4 proteins, whilst hesperidin treatment decreased the tumour numbers and increased the levels of Act-A and Smad4 in malignant tissues [7, 33]. By contrast, others have suggested that Act-A is oncogenic since the βA-subunit mRNA and protein alongside Act-A levels increased with CRC progression and linked directly with poor prognosis [17-20]. Act-A also enhanced the migration of malignant enterocytes *in vitro* through several Smad4-independent mitogenic pathways [10, 34]. Furthermore, TGF-β provoked CRC metastasis by elevating stromal Act-A *in vivo* and *in vitro*, whilst neutralisation of Act-A inhibited the TGF-β pro-metastatic effects [18]. Hence, the “TGF-β paradox” or “molecular switch” hypothesis has emerged to explicit the divergent actions of TGF-β and Act-A in colon carcinogenesis [10, 35, 36]. The theory, which is advocated by our findings, proclaims that Act-A may deter the advance of Smad4-positive tumours, whilst it could induce progression through non-canonical oncogenic pathways following the loss of Smad4 protein during the late stages of CRC [10, 35, 36].

Little is currently known about the roles of Act-AB and Act-B in CRC biology [7]. However, we have previously reported substantial decreases in the βB-subunit mRNA and protein that coincided with reductions in serum and intratumoral Act-AB, and their levels correlated negatively with the tumour numbers and sizes in a CRC rat model [7]. Serum Act-AB levels also declined in HER2-positive breast cancer patients, whereas a sudden acute rise in Act-A suggested cancer recurrence [37]. Moreover, Act-AB reduced cell viability in the IH-1 human myeloma cells [38] and antagonised Act-A actions in primary cultured rat hepatocytes by competing for their type II receptors, thus inhibited DNA synthesis [39]. While all activins commonly trigger Smad4 through ALK4, Act-B and -AB, but not Act-A, could also provoke Smad4-dependent actions via ALK7 [11, 12], which has been reported to potently inhibit cell proliferation and survival in several human cancer cell lines [40-42]. Act-AB could also mediate its actions using Smads 1/5/8 [38, 43, 44], the main mediators of the bone morphogenic proteins that act as tumour suppressors in CRC [45, 46].

Herein, the βB-subunit mRNA and protein alongside Act-B and Act-AB levels decreased in malignant compared with normal tissues. Moreover, the late-stage cancerous samples showed significantly lower βB-subunit mRNA and protein alongside Act-AB amounts, but not Act-B, compared with early-stage cancers. Act-AB also correlated inversely with Act-A and the tumour characteristics. Although the RSC and LSC neoplasms had equal amounts of Act-AB and Act-B during the early cancer stages, the former group had markedly lower Act-AB in the advanced stages. The Act-AB levels in the L-S4 malignant tissues were also substantially inferior to the N-S4 samples. Additionally, Act-AB increased the numbers of SW480 and HT29 cells in Sub-G1 and G0/G1-phases, whilst Act-B mainly induced G2/M-phase arrest in the HT29 cells. Moreover, the p21, p27, Casp-8 and Casp-3 proteins increased with concomitant declines in CCND1, CCND3, BCL2 and survivin in both cell lines with Act-AB. This study agrees with many prior reports and, altogether, suggest that Act-AB could be a Smad4-independent tumour suppressor protein in CRC [7, 37–39]. Our data further advocate that the dysregulation of activins could promote the initiation of CRC independent of anatomical site, whereas loss of Act-AB might contribute to the aggressiveness of right-sided neoplasms during the late cancer stages. Furthermore, we hypothesise that Act-AB could be a potential therapeutic target that might supress tumour progression through Smads 1/5/8 in Smad4-mutated colon cancers. However, more studies involving the different activin type I and II receptors together with Smads 1/5/8, are still needed to validate our hypothesis.

FS tightly controls the biological actions of activins by irreversible binding [7, 47]. Additionally, FS is pathologically altered in a variety of solid tumours and is thought to counteract the effects of Act-A [47]. However, both Act-A and FS increased with CRC progression in rats and their concentrations correlated together directly as well as with the numbers and sizes of tumours [7]. Moreover, the expression of FS was induced in malignant enterocytes following their co-culture with normal primary colonic fibroblasts and the protein enhanced cell migration and invasion [48], which also occurs in colon cancer cell lines treated with Act-A [10, 34]. Concurrently, FS increased markedly in desmoplastic invasion front of metastatic CRC, advocating a facilitatory role for the protein in tumour progression [21]. Increases in FS were also reported in other solid carcinomas, and the protein levels were associated with cancer progression and poor prognosis [49–51]. Prostate cancer cells also resisted the Act-A growth inhibitory effects by overproducing FS [52, 53].

This study showed stage-dependent expression of FS gene and protein in malignant tissues that was depicted by initial decreases during the early stages followed by a drastic upsurge in the late stages of cancer. Additionally, the cancerous tissues FS levels showed direct links with Act-A and the TNM categories, whilst correlated negatively with Act-AB. While FS treatment showed no effect on cell cycle in the SW480 cells, it provoked G1-arrest and enhanced the expression of p21, p27 and Casp-8 alongside decreased CCND1, CCND3 and survivin proteins in the HT29 cells. Our findings correlate with previous reports and suggest that the observed aberrant alterations in activins during colon carcinogenesis could be related to an atypical disequilibrium with FS [47, 54, 55]. In correspondence, we speculate that the concurrent decline in FS with increase in Act-A during the early CRC stages could suppress tumorigenesis by elevating the intratumoral free Act-A levels [52, 53]. In contrast, Act-A escalates in the late stages of cancer to compensate for FS overproduction that might promote cancer metastasis and progression by neutralising Act-A [21, 48]. Furthermore, there are no reports in the literature regarding the potential interactions between Act-B and -AB with FS during neoplastic diseases despite that the protein equally neutralises all activins [7, 47]. Hence, we also hypothesise that the deregulation of FS could provide a background for the observed decreases in both Act-B and Act-AB during colon carcinogenesis. However, co-treating colon cancer cell lines with diverse concentration ratio of the different mature activin proteins (A, B or AB) in the presence of follistatin (1:1, 2:1 and 1:2) are required to verify our suggestion.

In conclusion, activins and follistatin are pathologically altered in CRC and their deregulations were more pronounced in advanced right-sided tumours and with the loss of Smad4 protein, suggesting their important contribution to cancer development and aggressiveness. Moreover, our data suggest that the paradoxical actions of Act-A in CRC could be dependent on Smad4 protein expression, which is commonly lost during the late stages of malignancy. This study is also the first to report consistent decreases in Act-AB with CRC progression and the protein induced cell cycle arrest and increased the markers of apoptosis in the SW480 and HT29 cells, implying that it could act as a Smad4-independent tumour suppressor. On the other hand, FS showed biphasic stage-dependent expression, which might induce the observed abnormalities in colonic activins and subsequently cancer progression. However, further studies are still required to explore the complex connections between activins and their related proteins as well as to investigate their expression and actions in relation to tumour microsatellite status and consensus molecular subtypes to precisely explicate their roles in human CRC.

## Data Availability

The original contributions presented in the study are included in the article/Supplementary Material, further inquiries can be directed to the corresponding author.

## Supplementary Material

Supplementary Figure S1 Localisation of activin βA- and βB-subunits, follistatin, and Smad4 proteins by immunohistochemistry (IHC) in the FFPE (*n* = 90 patients) and fresh (*n* = 40 patients) cohorts of normal colonic tissues according to the clinical stages of cancer and tumour sidedness (Scale bar = 15 μm). Additionally, the expression of Smad4 protein is shown by immunofluorescence in the SW480 and HT29 cells (Scale bar = 8 μm).
