# Supplementary material for: Profiling Activins and Follistatin in Colorectal Cancer According to Clinical Stage, Tumour Sidedness and Smad4 Status
Source: Pathol Oncol Res. 2021 Nov 15;27:1610032. doi: 10.3389/pore.2021.1610032 (PMC8634429; doi:10.3389/pore.2021.1610032)
Supplement: Supplementary file 1 [file Table1.DOCX]

**Supplementary table 1.** The sequences of PCR primers used for the detection of human *β-actin*, *INHBA, INHBB, Acvr2A, Acvr2B, FST and Smad4* mRNAs in colon samples including the corresponding genes accession numbers and amplicon sizes.

| **Genes** | **Forward** | **Reverse** | **Amplicon size** |
| --- | --- | --- | --- |
| ***ACTB***  (NCBI: NM_001101.5) | 5’ ATC CCC CAA AGT TCA CAA TG 3’ | 5’ GTG GCT TTT AGG ATG GCA AG 3’ | 117 bp |
| ***INHBA***  (NCBI: NM_002192.4) | 5’ GAA GAG TGG GGA CCA GAA AGA G 3’ | 5’ TTT TAA AAG GCC CTG CTT TTC CTC 3’ | 134 bp |
| ***INHBB***  (NCBI: NM_002193.4) | 5’ GAG CGC GTT TCC GAA ATC ATC 3’ | 5’ CCT GGA CCA CAA ACA GGT TCT 3’ | 115 bp |
| ***Acvr2A***  (NCBI: NM_001278579.2) | 5’ AGG CTA ATG TGG TCT CTT GGA A 3’ | 5’ CCA ATC CTC TAG CCA TGG TTT CT 3’ | 63 bp |
| ***Acvr2B***  (NCBI: NM_001106.4) | 5’ GGA GTG CAT CTA CTA CAA CGC 3’ | 5’ TCC AGG CCG CTC TGG TT 3’ | 60 bp |
| ***FST***  (NCBI: NM_006350.5) | 5’ CAG TTC ATG GAG GAC CGC AG 3’ | 5’ CTT GTA CAG GAC CTG GCA GC 3’ | 84 bp |
| ***Smad4***  (NCBI: NM_005359.6) | 5’ GTG GCT GGT CGG AAA GGA TTT 3’ | 5’ GAT CAG GCC ACC TCC AGA GAC 3’ | 61 bp |
