# Supplementary figures and images for: Profiling Activins and Follistatin in Colorectal Cancer According to Clinical Stage, Tumour Sidedness and Smad4 Status
Source: Pathol Oncol Res. 2021 Nov 15;27:1610032. doi: 10.3389/pore.2021.1610032 (PMC8634429; doi:10.3389/pore.2021.1610032)

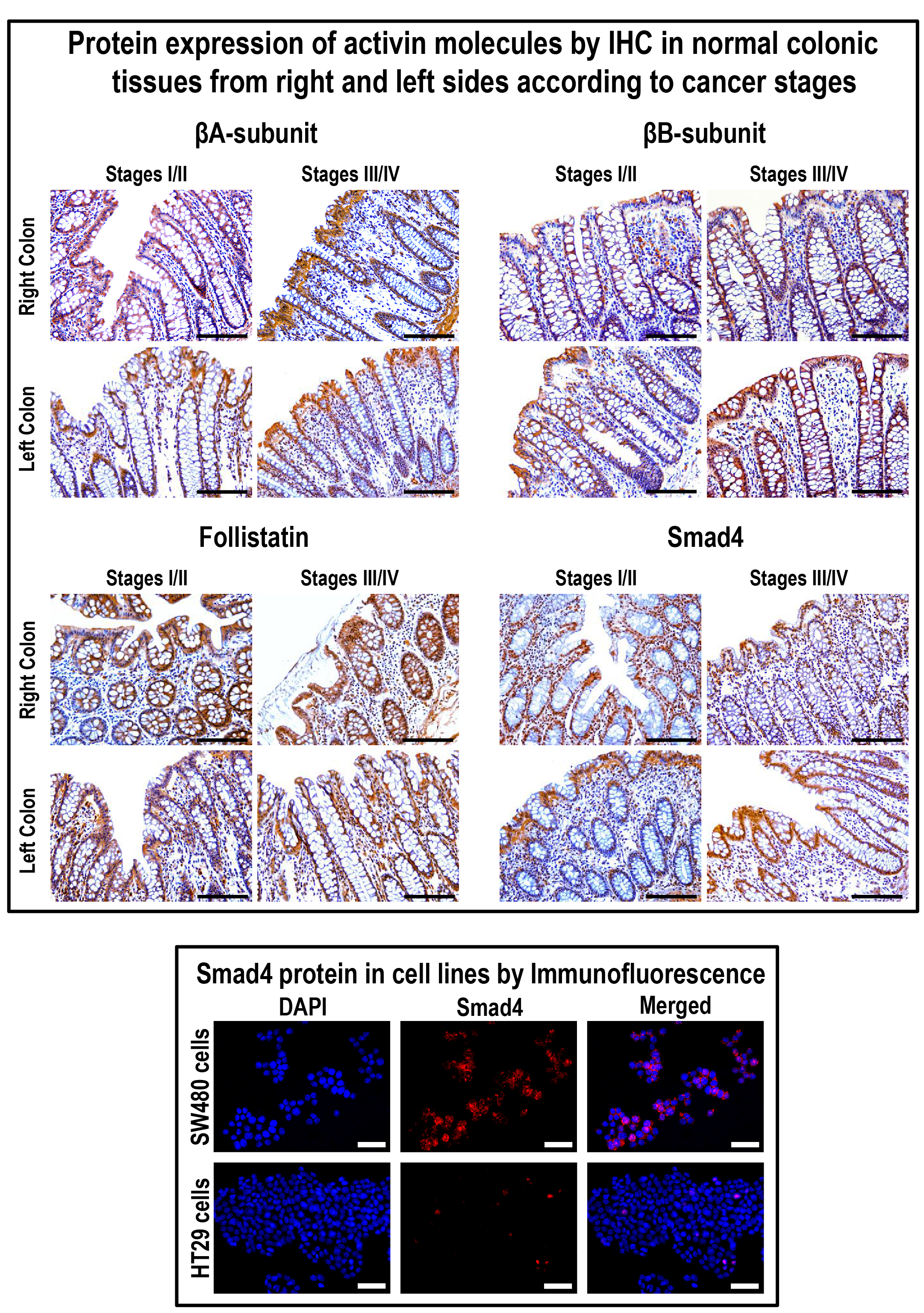

Supplement: Supplementary file 2 [file Image1.JPEG]
